# MCT4 drives HCC progression by activating MMPs and polarizing M2 macrophages

**DOI:** 10.3389/fimmu.2026.1777015

**Published:** 2026-07-01

**Authors:** Kaiyuan Zhang, Xiaochen Ni, Chuhang Wang, Jianing Guo, Wei Fan, Tao Sun, Tao Jiang, Guangji Zhang

**Affiliations:** 1 School of Basic Medical Sciences, Zhejiang Chinese Medical University, Hangzhou, Zhejiang, China; 2 Zhejiang Key Laboratory of Blood-Stasis-Toxin Syndrome, Zhejiang Chinese Medical University, Hangzhou, Zhejiang, China; 3 The Second Affiliated Hospital of Zhejiang Chinese Medical University, Hangzhou, Zhejiang, China; 4 Traditional Chinese Medicine “Preventing Disease” Wisdom Health Project Research Center of Zhejiang, Hangzhou, Zhejiang, China

**Keywords:** hepatocellular carcinoma, M2 macrophage polarization, matrix metalloproteinases, MCT4, tumor immune microenvironment

## Abstract

**Background:**

Hepatocellular carcinoma (HCC) remains a leading cause of cancer-related mortality worldwide, with a poor survival rate despite advances in therapy. Metabolic reprogramming, particularly involving lactate transport, plays a critical role in HCC progression. Monocarboxylate transporter 4 (MCT4) is a key lactate exporter often overexpressed in cancers, yet its precise role in HCC pathogenesis and immune modulation remains incompletely defined.

**Methods:**

We integrated bioinformatic analyses of multiple datasets (TCGA-LIHC, GSE46408, GSE36411) with experimental validation in HCC cell lines (Huh7, MHCC97-H) and a murine xenograft model. Functional assays including wound healing, Transwell invasion, Western blot, and flow cytometry were employed. Immune cell infiltration and polarization were analyzed via CIBERSORT and single-cell RNA sequencing data (GSE282701).

**Results:**

MCT4 was identified as a prognostic hub gene among lactate metabolism-related genes (LMRGs) and was significantly upregulated in HCC tissues, correlating with advanced tumor stage and poor survival. Gene Set Enrichment Analysis (GSEA) revealed a strong association between MCT4 expression and matrix metalloproteinase (MMP) pathways. *In vitro*, MCT4 knockdown downregulated MMP1, MMP2, and MMP9 expression and suppressed HCC cell migration and invasion. Furthermore, high MCT4 expression was linked to an immunosuppressive tumor microenvironment characterized by M2 macrophage polarization. *In vivo*, MCT4 knockdown inhibited tumor growth and reduced infiltration of CD206^+^ M2 macrophages.

**Conclusions:**

Our findings demonstrate that MCT4 drives HCC progression through two complementary mechanisms: enhancing MMP-mediated invasion and metastasis, and promoting immunosuppressive M2 macrophage polarization. These results nominate MCT4 as a promising therapeutic target for restoring antitumor immunity and inhibiting metastasis in HCC.

## 1 Introduction

Hepatocellular carcinoma (HCC) accounts for approximately 80% of primary liver malignancies and ranks as the sixth most commonly diagnosed cancer and the third leading cause of cancer-related deaths worldwide (1, 2). Although significant advances have been made in recent years with targeted therapies, such as anti-angiogenic tyrosine kinase inhibitors, and immunotherapies including anti-PD-1 agents, the five-year survival rate of HCC patients remains below 20% (3). Therefore, there is an urgent need to further explore the pathogenesis of HCC in order to identify suitable therapeutic targets.

Monocarboxylate transporter 4 (MCT4, also known as solute carrier family 16 member 3 or SLC16A3) belongs to the proton-coupled monocarboxylate transporter family and was identified in 1976 as a key lactate transporter (4). It facilitates the transport of short-chain glycolytic metabolites such as pyruvate and lactate, thereby helping to maintain intracellular pH and sustain glycolysis (5). MCT4 has been associated with poor prognosis in multiple cancer types (6). Clinical data indicate that MCT4 expression is elevated in HCC tissues compared with adjacent normal liver tissues, and its expression correlates significantly with larger tumor size, advanced TNM stage, and unfavorable patient outcomes (7–9). Despite these insights, the specific role of MCT4 in HCC progression and its influence on the tumor immune microenvironment (TIME) remain incompletely characterized.

**Figure 1:**
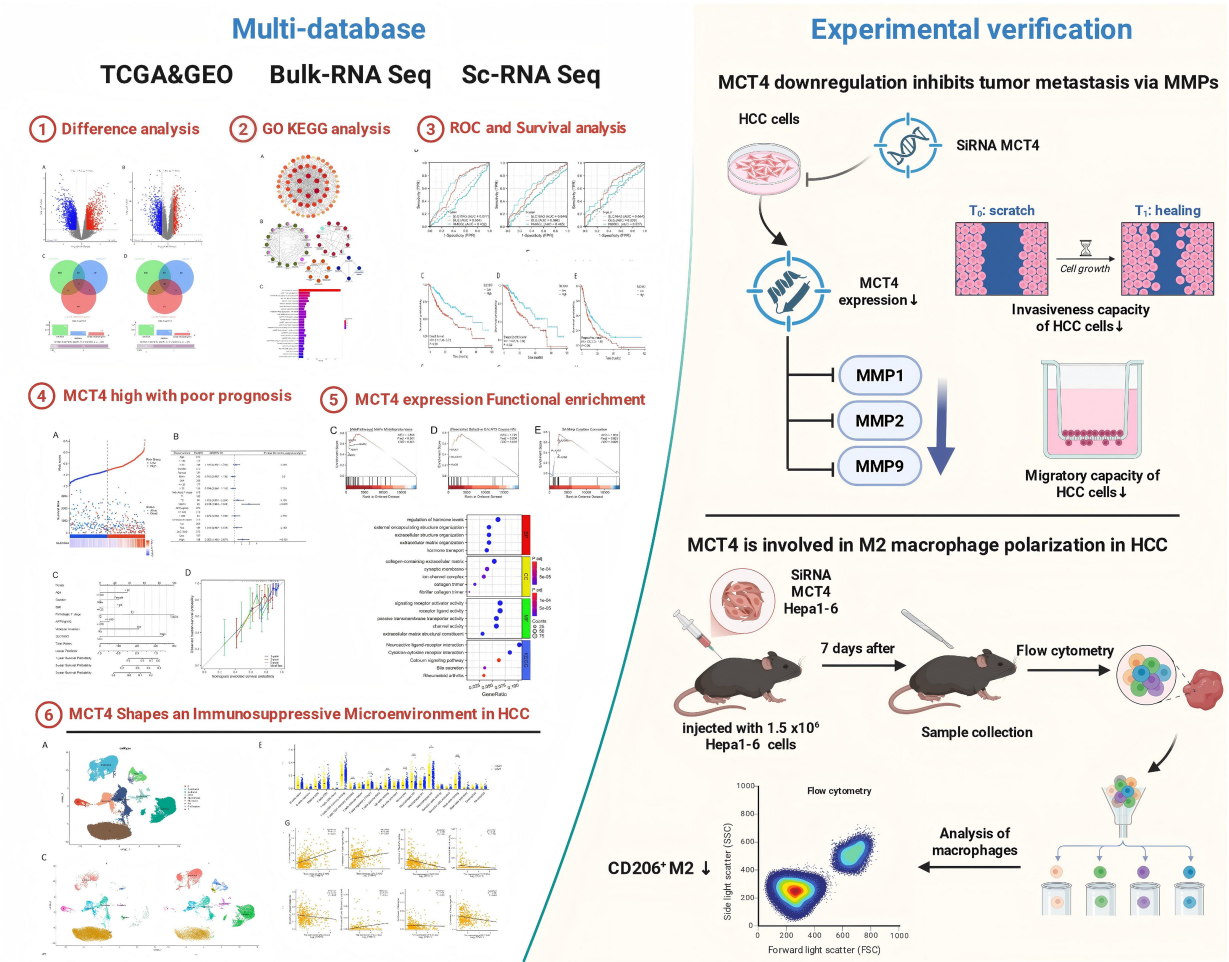
Flow chart. First, bioinformatic analyses were conducted using multiple datasets (TCGA-LIHC, GEO, single-cell RNA-seq) to identify LMRGs, screen hub genes, and confirm MCT4 as a key prognostic gene for HCC. Second, *in vitro* experiments were performed: MCT4 was knocked down in HCC cell lines, and functional assays such as wound healing, Transwell, and Western blot verified that MCT4 downregulation reduces HCC cell migration/invasion and inhibits MMP expression. Third, *in vivo* validation was carried out using a murine HCC xenograft model: MCT4-silenced cells were implanted, tumor growth was monitored, and flow cytometry analyzed immune infiltration, especially M2 macrophage polarization. Finally, the study concluded that MCT4 promotes HCC progression by enhancing MMP-mediated invasion and shaping an immunosuppressive microenvironment via M2 polarization.

We evaluated the clinical relevance of MCT4 in HCC. Our study integrated bioinformatic analyses and experimental validation, revealing that MCT family members are not only involved in HCC progression but also closely associated with the expression of the MMP family proteins, thereby influencing HCC cell invasion and metastasis. Furthermore, we demonstrated that MCT4 contributes to remodeling the immune microenvironment of HCC, particularly by promoting M2 macrophage polarization. These findings underscore the multifaceted role of MCT4 in HCC: it facilitates tumor invasion and metastasis through upregulation of the MMP family genes, while simultaneously enhancing M2 macrophage polarization within the tumor immune microenvironment. This metabolic reprogramming mechanism highlights the link between glycolytic metabolism and immune evasion in HCC, suggesting MCT4 as a potential therapeutic target worthy of further investigation. **Figure 1** provides an overview of the study design.

## 2 Methods

### 2.1 Exploration of microarray data and lactate metabolism genes

The microarray dataset GSE46408 (containing 6 HCC samples and 6 normal samples) and GSE36411 (containing 12 HCC samples and 20 normal samples) were retrieved from the GEO (https://www.ncbi.nlm.nih.gov/). Clinical information and RNA-seq data were obtained from the LIHC cohort of The Cancer Genome Atlas (TCGA). For the training cohort, only HCC tumor samples with complete clinical information, including survival time, survival status, age, and gender, were included. When paired samples were available, only one tumor sample per patient was retained. Samples lacking complete clinical information were excluded. A total of 377 LIHC tumor samples were included in the training cohort. Lactate metabolism genes were downloaded from GeneCards (https://www.genecards.org/). For clarity and consistency, the gene symbol SLC16A3 was used in the bioinformatic and transcriptomic analyses, whereas MCT4 was used in the experimental sections to refer to the encoded protein.

### 2.2 Acquisition of lactate metabolism related differentially expressed genes

The transcriptomic datasets GSE46408 and GSE36411 were analyzed using the Xiantao platform (https://www.xiantaozi.com/) for differentially expressed gene identification. A threshold of *P* < 0.05 and |log2FC|≥1 was applied for screening DEGs in the GSE46408 and GSE36411 datasets. To identify LMRGs in HCC, Venny 2.1 (https://bioinfogp.cnb.csic.es/tools/venny/index.html) was used to overlap lactate metabolism-related genes from GeneCards with the identified DEGs from GSE46408 and GSE36411. Finally, the online tool Xiantao Academic (https://www.xiantaozi.com/) was utilized for visualizing the volcano plot of the identified LMRGs.

### 2.3 Protein-protein interaction and enrichment analysis

PPI analysis of LMRGs in HCC was conducted using STRING (http://www.string-db.org), with a minimum required interaction score ≥ 0.4 set as the confidence threshold for protein interactions (10). The results were imported into Cytoscape (http://www.Cytoscape.org) (11) for visualization. Gene Ontology (GO) enrichment analysis was performed using ClueGO, while Kyoto Encyclopedia of Genes and Genomes (KEGG) enrichment analysis was conducted through the DAVID database (https://davidbioinformatics.nih.gov/). A free online platform Wei Sheng Xin (https://www.bioinformatics.com.cn) was utilized for visualizing the results. *P* < 0.05 was considered statistically significant.

### 2.4 Analysis of hub genes

Hub genes were identified using the CytoHubba plugin in Cytoscape. The top 10 Down-regulated hub LMRGs were determined using seven algorithms: Maximum Clique Centrality (MCC), Maximum Neighborhood Component (MNC), Degree, Closeness, Stress, Betweenness, and Radiality. The genes identified by these algorithms were intersected to obtain the final set of critical genes. Subsequently, a co-expression network of these hub genes was constructed using GeneMANIA (http://www.Genemania.org/) (12).

### 2.5 Validation of the expression of hub LMRGs and their survival analysis

The mRNA expression of the identified hub genes was validated in the TCGA-LIHC. Comparisons between groups were performed using t-test. *P*-value < 0.05 was considered significant. TCGA-LIHC patients were divided into two distinct cohorts according to the median expression levels of the hub LMRGs, with the low-expression group comprising the bottom 50% and the high-expression group encompassing the top 50%. Survival outcomes were assessed using Kaplan-Meier and Cox regression analyses. The Xiantao tool, specifically the “Survival” package, was utilized to compare overall survival (OS), disease-specific survival (DSS), and progression-free interval (PFI) between these two expression groups.

### 2.6 Construction of the receiver operating characteristic model

ROC curves were applied to evaluate the diagnostic potential of hub LMRGs (SLC16A3, GLS, and HMGCL) in the TCGA-LIHC dataset. The ROC analysis was conducted and visualized using the Xiantao tool with the “pROC,” “timeROC,” and “ggplot2” packages. A gene’s diagnostic value increases as the area under the ROC curve (AUC) approaches “1.”

### 2.7 Univariate logistic regression analysis

Univariate logistic regression analysis was carried out using the Xiantao tool with the “stats” package. HCC patients were categorized into two groups based on the median mRNA expression of SLC16A3, with the low-expression group acting as the reference. Logistic regression models were employed to evaluate the association between SLC16A3 expression levels and various clinicopathologic features.

### 2.8 Cox regression analysis and prognostic model generation

The proportional hazards hypothesis was tested using the Xiantao tool with the “survival” package, followed by univariate and multivariate Cox regression analyses to evaluate the independent prognostic significance of high SLC16A3 expression and various clinicopathologic factors in the OS of HCC patients. Clinicopathologic variables with *P* < 0.10 in the univariate Cox regression analysis were included in the multivariate Cox regression model to avoid excluding potentially relevant prognostic factors at the screening stage. In the final statistical analyses, *P* < 0.05 was considered statistically significant. Forest plots, risk score curves, and survival point charts were generated using the Xiantao tool with the “ggplot2” package. Based on the independent prognostic factors identified through multivariate Cox regression analysis, a nomogram model was developed using the Xiantao tool with the “rms” package to predict the 1-, 3-, and 5-year OS of HCC patients. Calibration analysis and visualization were also performed using the same tool and package, illustrating the discrepancies between the predicted probabilities and the observed outcomes at various time points.

### 2.9 Gene sets enriched in SLC16A3 expression phenotype

In the analysis of TCGA RNA-seq data, HCC patients were stratified according to the median mRNA expression level of SLC16A3. Patients with SLC16A3 expression below or equal to the median were assigned to the low-expression group, corresponding to the bottom 50% of the cohort, whereas patients with expression above the median were assigned to the high-expression group, corresponding to the top 50%. This median-based cutoff was selected to avoid arbitrary threshold determination and to generate balanced groups for differential expression and enrichment analyses. Differential expression analysis was performed using the Xiantao tool based on the processed expression data available from the platform, with the “DESeq2” and “limma” packages used in the analytical workflow. Genes with an absolute |log2FC| ≥1 and *P* < 0.05 were considered significant DEGs and included in subsequent analyses. Volcano plots were generated using the Xiantao tool with the “ggplot2” package. To explore differences in biological functions and signaling pathways between the high- and low-expression groups, the identified DEGs underwent GO and KEGG enrichment analyses using the Xiantao tool with the “clusterProfiler” package. Enrichment terms with an adjusted p-value < 0.05 were deemed significant. For Gene Set Enrichment Analysis (GSEA), the “c2.cp.all.v2022.1.Hs.symbols.gmt [All Canonical Pathways]” dataset from the Molecular Signatures Database (MSigDB) was used as the reference gene set. Terms with a false discovery rate (FDR) < 0.25 and an *P*-value < 0.05 were considered significantly enriched. The results of the GO, KEGG, and GSEA enrichment analyses were visualized using the Xiantao tool with the “ggplot2” package. Correlation between SLC16A3 and various DEGs was implemented by Spearman analysis. Visualization was performed using Xiantao tool.

### 2.10 Cell culture and treatment

The Huh7 (CL-0120), Hepa1-6 (CL-0105), and MHCC97-H (TCH-C258) cell lines were procured from Wuhan Pricella Biotechnology Co., Ltd. and Haixing Biotechnology Co., Ltd., respectively. Cell identity was verified by short tandem repeat profiling provided by the suppliers or performed according to standard cell bank quality-control procedures. Both cell lines were cultured in DMEM medium supplemented with 10% fetal bovine serum according to the manufacturers’ protocols. All cultures were maintained at 37 °C in a humidified atmosphere containing 5% CO_2_. The culture medium was refreshed every 48–72 hours, and cells were subcultured at 1:3 ratio upon reaching 90% confluency. Cell line authentication and mycoplasma contamination testing were performed regularly to ensure culture purity. Huh7 and MHCC97-H human HCC cell lines were used for *in vitro* knockdown, migration, invasion, RT-qPCR, and Western blot (WB) experiments. Huh7 is a well-differentiated human HCC cell line, whereas MHCC97-H is a highly metastatic human HCC cell line. Hepa1-6, a murine hepatoma cell line derived from C57L mice and commonly used in C57BL/6J mouse models, was used for *in vivo* xenograft experiments.

### 2.11 Wound healing assay

Wound healing assays were performed using Huh7 and MHCC97-H cells. Briefly, cells were plated in 6-well plates and allowed to reach near-confluency (90–100%). A uniform scratch was introduced in the monolayer using a sterile 200 µL pipette tip. After gently washing with PBS to remove dislodged cells, the medium was replaced with serum-free DMEM. Cells were then cultured for 48 h, and images of the wound areas were acquired at 0, 24, and 48 h using a Leica microscope (Leica Microsystems, Germany). The extent of wound closure was quantified with ImageJ software (National Institutes of Health, USA).

### 2.12 Transwell invasion assays

Cell migration was assessed using Transwell chambers with 8-μm pore inserts (Corning Life Science, NY, USA). Briefly, 1×10^5^ Huh7 or MHCC97-H cells were suspended in 200 μL serum-free medium and added to the upper chamber, while the lower compartment contained 600 μL complete medium with 10% FBS as a chemoattractant. Following 48 h of incubation, cells that had migrated to the lower surface were fixed with 4% paraformaldehyde, stained with crystal violet, and imaged. The number of migrated cells was determined using ImageJ software (National Institutes of Health, USA).

### 2.13 RNA interference

Gene silencing was achieved using small interfering RNA (siRNA) at a working concentration of 50 nM. Huh7, MHCC97-H, and Hepa1–6 cell lines were transfected with the siRNA complexes using Lipofectamine™ 2000 (Invitrogen, USA) following the manufacturer’s guidelines. Cells were collected for subsequent analysis at 48 hours after transfection. The specific siRNA sequences used in this study are provided in **Supplementary Table 1**.

### 2.14 Protein extraction and WB analysis

Total protein was extracted from cells or tissues using RIPA lysis buffer (Beyotime Biotechnology, China) supplemented with protease and phosphatase inhibitors (Applygen Technologies, China). Protein concentration was determined by the bicinchoninic acid (BCA) method (Thermo Fisher Scientific, USA). Proteins were denatured, resolved by SDS-PAGE, and transferred to a nitrocellulose membrane (Millipore, USA). After blocking with 5% bovine serum albumin for 1 h at room temperature, the membranes were probed with primary antibodies at 4 °C overnight. Following three washes, membranes were incubated with corresponding secondary antibodies for 1 h at room temperature. Protein signals were detected using an Odyssey 290 infrared imaging system (LI-COR Biosciences, USA), and band intensities were quantified with ImageJ software (National Institutes of Health, USA). GAPDH was used as a loading control. Antibody details are provided in **Supplementary Table 2**.

### 2.15 RNA extraction and RT-qPCR analysis

Total RNA was extracted using Tri-Reagent in accordance with standard phenol-chloroform-based procedures. Reverse transcription was carried out with oligo primers and reverse transcriptase under the thermal conditions recommended by the manufacturer. For quantitative real-time PCR, SYBR Green master mix was employed on a real-time PCR detection system, with thermal cycling conditions consisting of an initial denaturation at 95 °C for 30 seconds, followed by 40 cycles at 95 °C for 5 seconds and 60 °C for 30 seconds. Primers specific to the target genes (listed in **Supplementary Table 3**) were used for amplification. Relative mRNA abundances were determined by normalizing to β-actin (ACTB) and applying the 2^−ΔΔCt^ method. Three technical replicates were included for each experimental group.

### 2.16 Establishment of mouse HCC xenograft model

Female C57BL/6J mice (6–8 weeks old, 18–22 g) were acclimatized for one week under SPF conditions at Zhejiang Chinese Medical University. A total of six mice were included in each group: the control group received subcutaneous injections of 2×10^6^ Hepa1–6 cells, while the experimental group received 2×10^6^ Hepa1–6 shMCT4 cells. After inoculation, mice were maintained for four weeks under standard conditions, and tumor dimensions were regularly recorded. All animal procedures were approved by the Institutional Animal Care and Use Committee of Zhejiang Chinese Medical University and conducted in accordance with relevant guidelines.

### 2.17 Preparation of single-cell suspensions from tumor tissues and flow cytometry

Upon completion of the study, mice were euthanized by cervical dislocation. Tumor tissues were excised, weighed, and placed in ice-cold PBS. The tissues were minced into approximately 1 mm³ fragments and digested with collagenase IV (1 mg/mL) at 37 °C for 40 minutes, with vortexing every 10 minutes. The resulting cell mixtures were filtered through 70 μm and 40 μm cell strainers sequentially. After centrifugation at 300g for 5 minutes, the cell pellets were treated with 1× red blood cell lysis buffer for 5 minutes at room temperature in the dark. The reaction was terminated with excess PBS, and cells were resuspended in PBS containing 2% FBS for counting. To block nonspecific Fc receptor binding, cells were incubated with anti-mouse CD16/32 antibody on ice for 10 minutes, followed by staining with fluorochrome-conjugated antibodies for 30 minutes on ice in the dark. The following antibodies were shown in **Supplementary Table 2**. After staining, cells were washed twice with FACS buffer and resuspended in PBS containing DAPI for live-dead discrimination. Samples were analyzed immediately using a flow cytometer, and data were processed with FlowJo software.

### 2.18 Statistical analysis

All statistical analyses were performed using R software (version 4.4.1). For comparisons between two groups, Student’s t-test was used for normally distributed data, whereas the Wilcoxon rank-sum test was applied for non-normally distributed data. For comparisons among multiple groups, one-way analysis of variance (ANOVA) or the Kruskal-Wallis test was used as appropriate. Correlation analyses were conducted using either Pearson’s or Spearman’s correlation test depending on data distribution and variable characteristics. Survival differences were evaluated using the Kaplan-Meier method and compared with the log-rank test. Univariate and multivariate prognostic analyses were performed using the Cox proportional hazards regression model. Unless otherwise specified, differential expression analysis and other high-throughput data analyses were conducted using the corresponding R packages with their recommended statistical frameworks. Data visualization was performed using the ggplot2 and ggpubr packages. *P* < 0.05 was considered statistically significant.

## 3 Results

### 3.1 Identification of LMRGs in HCC

**Figure 2:**
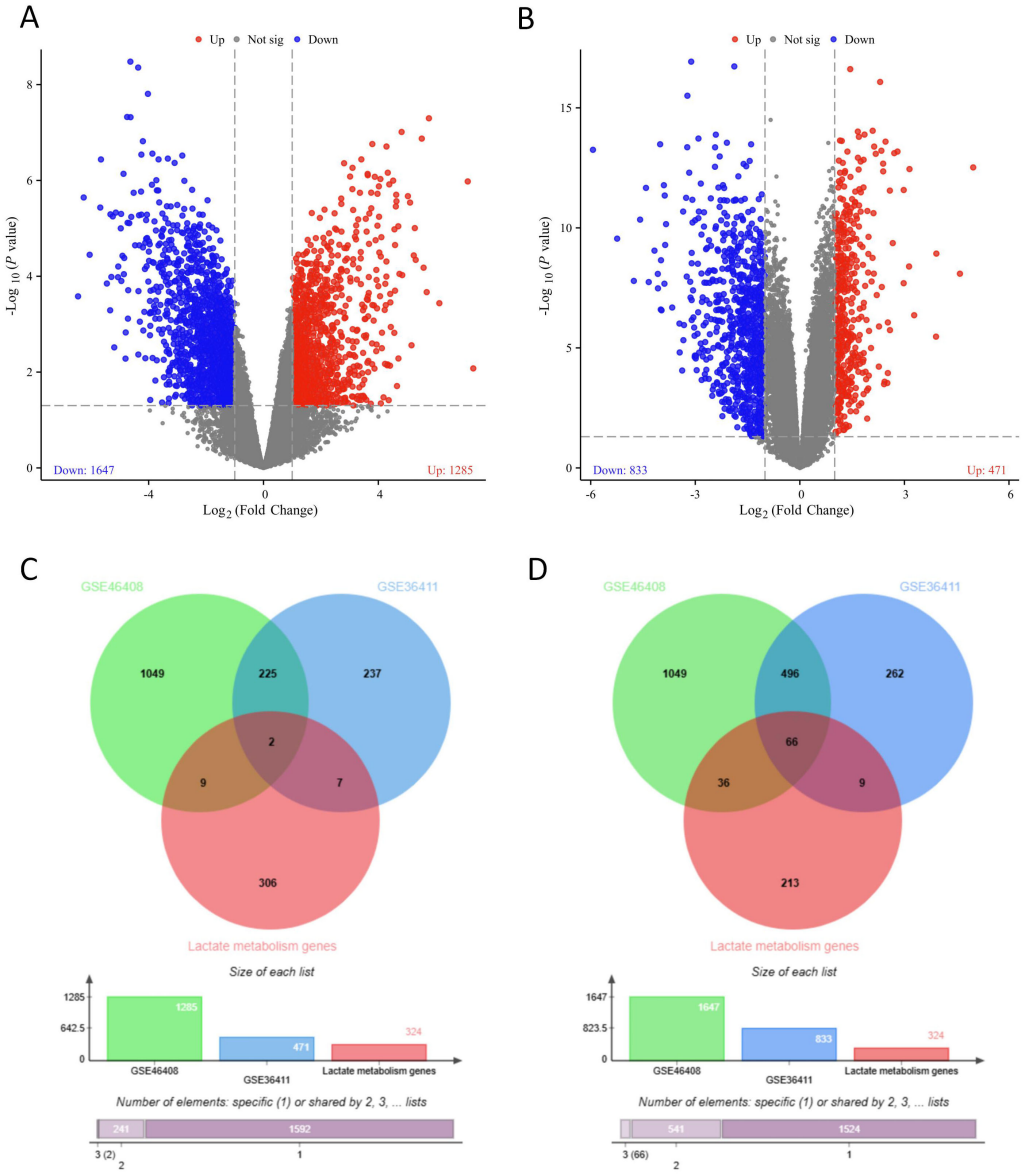
Identification of LMRGs. **(A)** The volcano plot of GSE46408. **(B)** The volcano map of GSE36411. **(C)** The venn diagram shows the intersection between upregulated LMRGs. **(D)** The venn diagram shows the intersection between downregulated LMRGs. Genes with *P* < 0.05 were considered significantly differentially expressed.

Based on the established criteria, we screened out 1285 upregulated and 1647 downregulated DEGs from the GSE46408, and 471 upregulated and 833 downregulated DEGs from the GSE36411 (**Figures 2A, B**). Additionally, we identified 324 genes with a lactate metabolism-related correlation score exceeding 20. By intersecting the DEGs with the lactate metabolism correlation genes, we obtained a total of 2 upregulated (**Figure 2C**) and 66 downregulated LMRGs (**Figure 2D**).

### 3.2 PPI analysis and enrichment analysis in LMRGs

**Figure 3:**
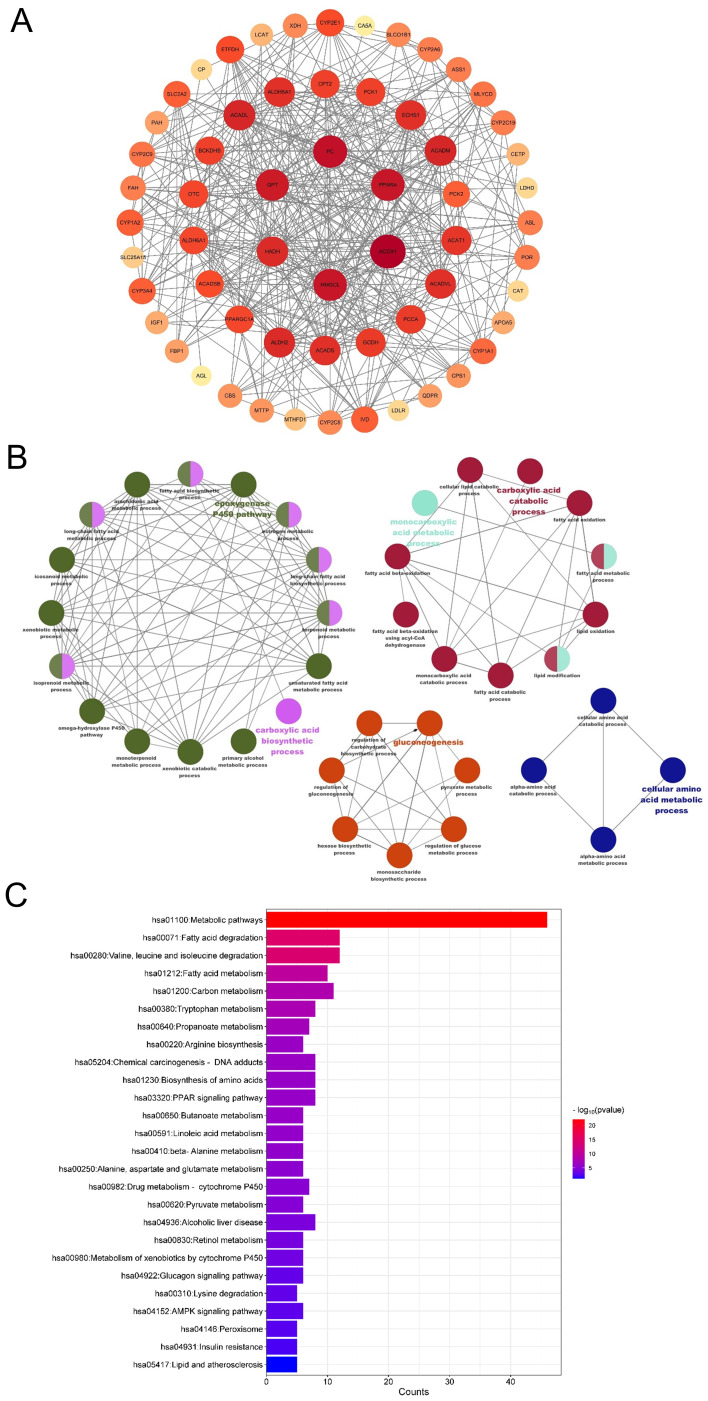
Explore the biological functions of LMRGs. **(A)** Protein-protein interaction (PPI) network of LMRGs generated using STRING and visualized with Cytoscape. Node color indicates degree value, defined as the number of direct connections between a given gene/protein node and other nodes in the network. A higher degree value indicates greater connectivity and potential centrality within the PPI network, with colors changing from yellow to red to represent increasing degree values. **(B)** Gene Ontology (GO) enrichment analysis of LMRGs performed using ClueGO. **(C)** Kyoto Encyclopedia of Genes and Genomes (KEGG) pathway enrichment analysis of LMRGs. For enrichment analyses, P < 0.05 was considered statistically significant.

The PPI analysis revealed that downregulated DEGs, including ACADS, HMGCL, ACAT1, ACOX1, BCKDHB, PC, and GPT among the LMRGs, demonstrated strong interaction patterns, with their colors ranging from yellow to red, indicating an increasing Degree value (**Figure 3A**). The ClueGO results showed significant enrichment of these 68 LMRGs in signaling pathways such as gluconeogenesis, carboxylic acid biosynthetic process, carbokytic acid catabolic process, and cellular amino acid metabolic process (**Figure 3B**). Additionally, the KEGG enrichment analysis indicated that these LMRGs were enriched in pathways related to metabolic pathways, carbon metabolism, and Valine, leucine and isoleucine degradation (**Figure 3C**).

### 3.3 Hub LMRGs selection and analysis

**Figure 4:**
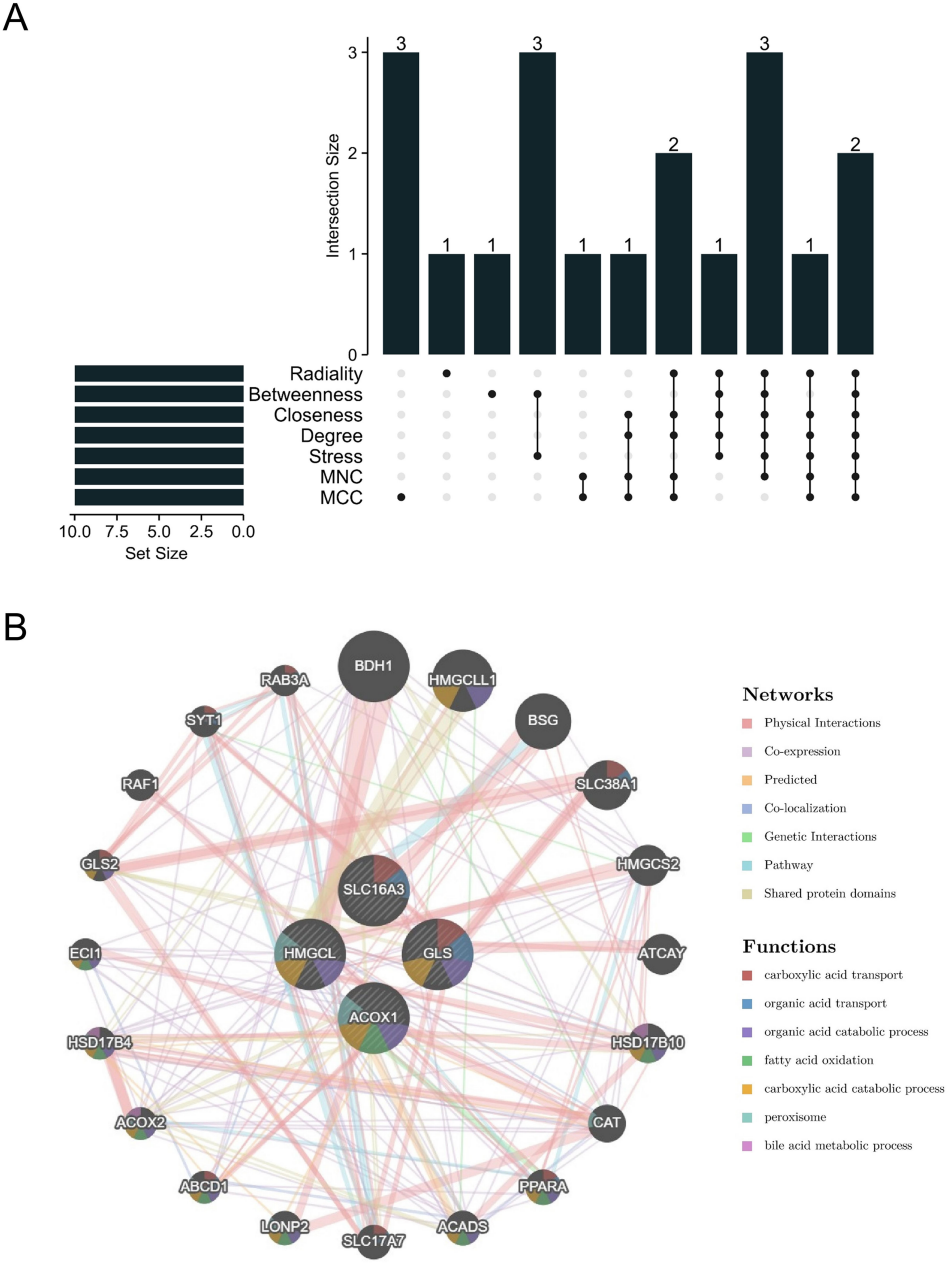
Co-expression network of LMRGs hub genes. **(A)** UpSet plot showing the 2 overlapping hub downregulated LMRGs identified by seven PPI algorithms. **(B)** Interaction network of the 4 hub LMRGs demonstrated through GeneMANIA.

To further identify hub genes among the LMRGs in HCC, we employed seven algorithms to select the top 10 hub genes. By intersecting the results from these seven algorithms, we ultimately identified 2 downregulated hub LMRGs: ACOX1 and HMGCL (**Figure 4A**, **Table 1**). SLC16A3 and GLS as the upregulated hub LMRGs. Then, we constructed a gene interaction network centered on 4 hub LMRGs. The analysis indicated that ACOX1, HMGCL, SLC16A3, and GLS exhibited strong interactions, with functions related to carboxylic acid transport, carboxylic acid catabolic process, organic acid transport, and fatty acid oxidation (**Figure 4B**).

**Table 1:** The Top 10 hub genes rank in cytoHubba.

MCC	MNC	Stress	Degree	Closeness	Betweenness	Radiality
ACADS	ACOX1	PPARA	ACOX1	ACOX1	PPARA	ACOX1
HMGCL	PPARA	GPT	PC	PC	GPT	PPARA
ACAT1	PC	ACOX1	PPARA	PPARA	ACOX1	GPT
ACOX1	HMGCL	PC	HMGCL	GPT	PC	PC
ECHS1	GPT	ALDH2	GPT	HMGCL	ALDH2	ACADL
ACADM	ACADL	OTC	ACADL	ACADL	SLC2A2	PCK1
ACADL	ACADM	SLC2A2	ACADM	ACADM	OTC	HADH
BCKDHB	HADH	CYP2E1	HADH	HADH	ALDH5A1	ACADM
ACADVL	ACADS	HMGCL	ALDH2	ALDH2	HMGCL	ALDH2
HADH	ACADVL	HADH	ACADS	ACADS	CYP2E1	HMGCL

### 3.4 SLC16A3 expression is elevated in HCC and associated with worse prognosis

**Figure 5:**
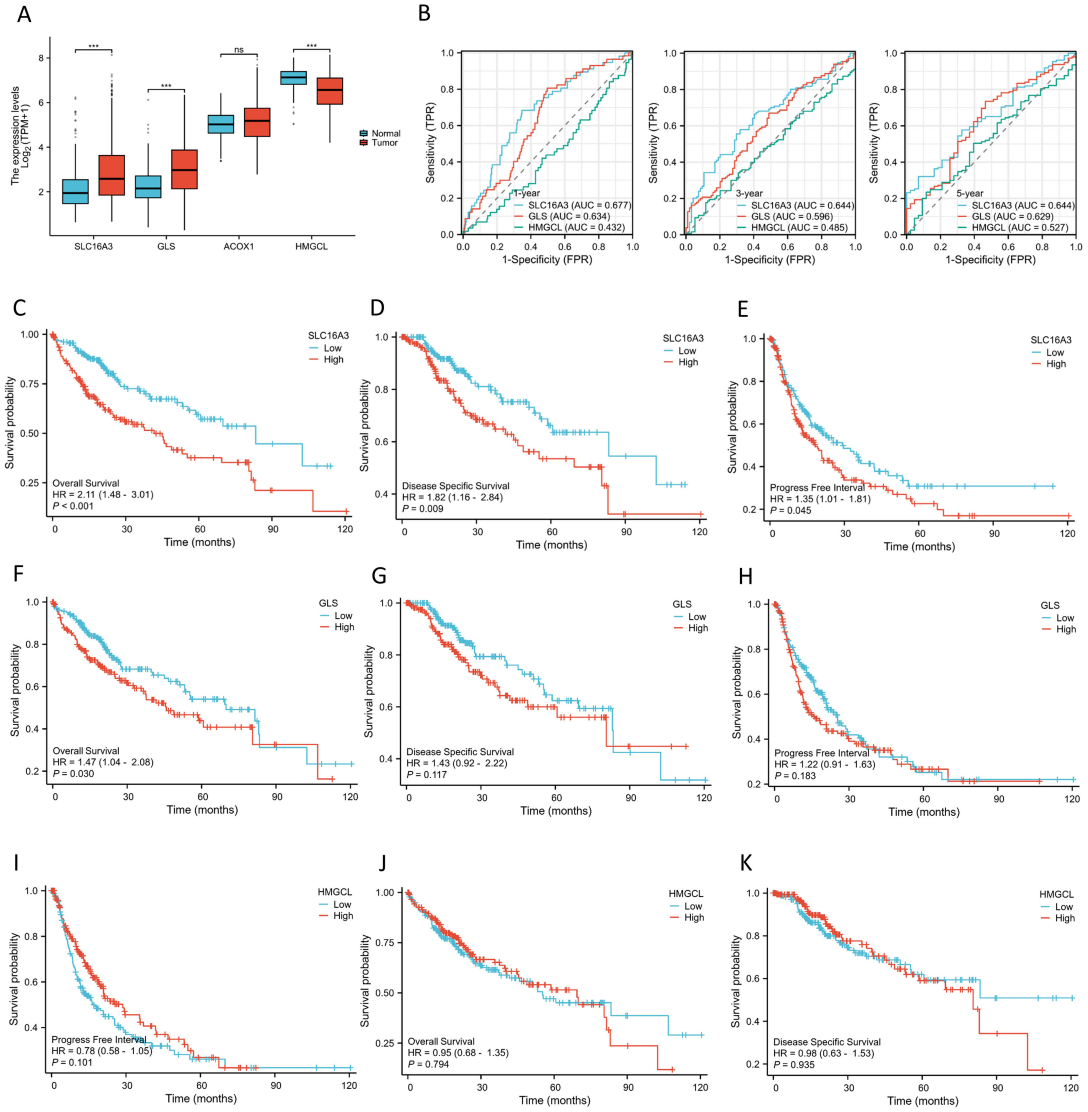
Further exploration of hub LMRGs in TCGA-LIHC. **(A)** The expression level of SLC16A3, GLS, ACOX1, and HMGCL in TCGA-LIHC. **(B)** Time-dependent ROC curves for 4 hub LMRGs expression in TCGA-LIHC. Survival curves of SLC16A3 for OS **(C)**, DSS **(D)** and PFI **(E)** in TCGA-LIHC. Survival curves of GLS for OS **(F)**, DSS **(G)** and PFI **(H)** in TCGA-LIHC. Survival curves of HMGCL for OS **(I)**, DSS **(J)** and PFI **(K)** in TCGA-LIHC. For panel A, differences between groups were analyzed using the Wilcoxon rank-sum test. Survival differences were analyzed using the Kaplan-Meier method with the log-rank test. Diagnostic performance was evaluated by ROC curve analysis, and the AUC was calculated. *P* < 0.05 was considered statistically significant.

To further validate the differential expression of 4 hub LMRGs between groups, we performed inter-group gene expression analysis using the TCGA-LIHC validation set (**Figure 5A**). While ACOX1 showed no significant differences between the groups (*P*>0.05), SLC16A3, GLS, and HMGCL were significantly overexpressed in the HCC group (*P* < 0.05). Then, Time-dependent ROC analysis demonstrated the most favorable predictive efficacy of SLC16A3 for OS at 1-year (AUC = 0.677), 3-years (AUC = 0.644), and 5-years (AUC = 0.644) (**Figure 5B**). KM analysis demonstrated that HCC patients only with high SLC16A3 expression exhibited poor OS, DSS and PFI (**Figures 5C–E**). On the contrary, KM analysis showed that the expression levels of GLS and HMGCL were at a disadvantage in predicting OSS, DSS and PFI (**Figures 5F–K**). These findings suggested that SLC16A3 expression is elevated in HCC and associated with worse prognosis, warranting further investigation.

### 3.5 High SLC16A3 expression associated with malignant phenotypes of HCC

**Figure 6:**
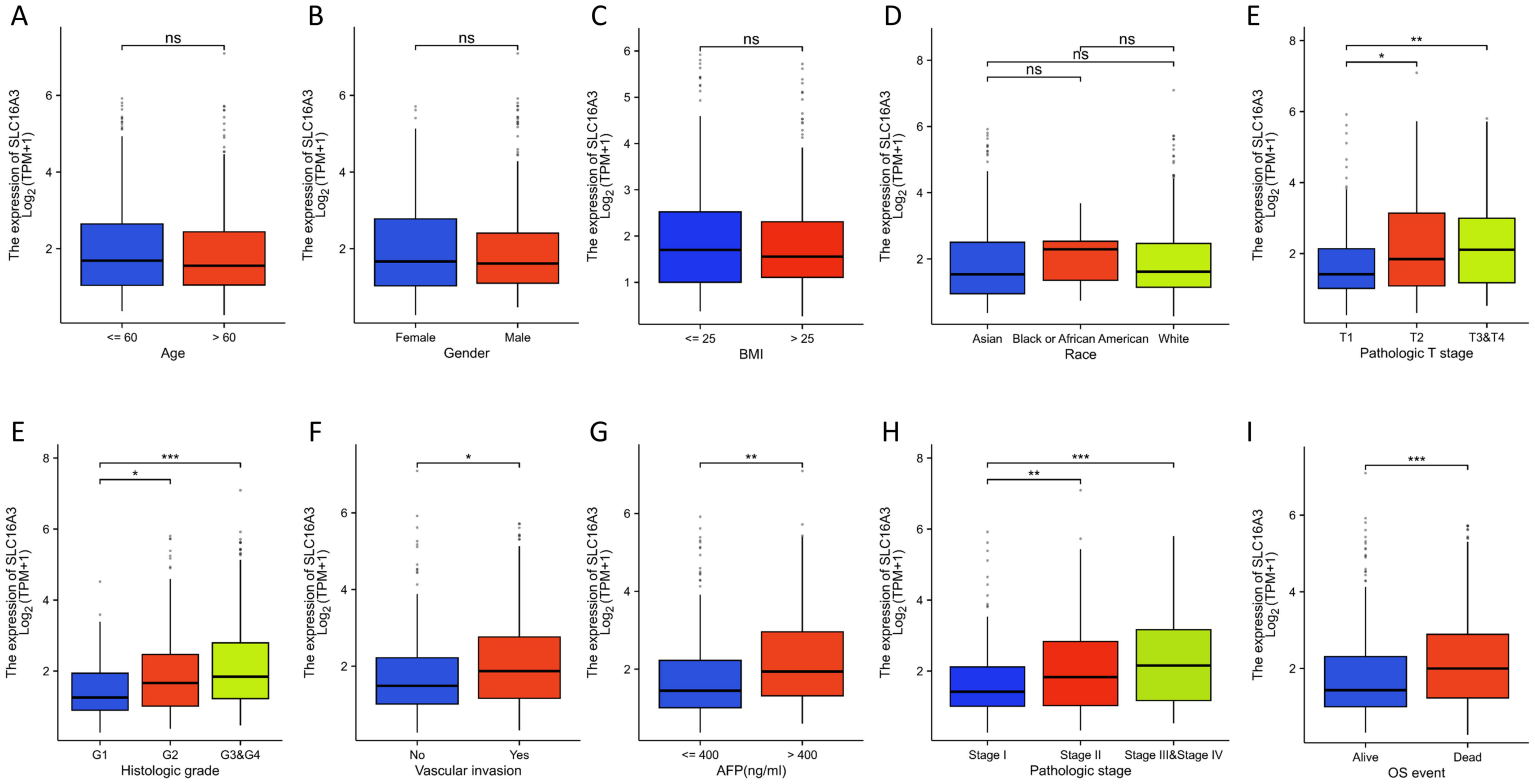
Associations between SLC16A3 expression and various clinicopathologic characteristics in HCC. **(A)** Age. **(B)** Gender. **(C)** BMI. **(D)** Race. **(E)** Pathologic T stage. **(F)** Histologic grade. **(G)** Vascular invasion. **(H)** AFP. **(I)** Pathologic stage. **(J)** OS event. Differences between groups were analyzed using the Wilcoxon rank-sum test or the Kruskal-Wallis test, as appropriate. ^*^P < 0.05, ^**^P < 0.01, ^***^P < 0.001, ns, not significant.

We investigated the association between SLC16A3 expression and various clinicopathologic characteristics of HCC patients in the TCGA database. SLC16A3 expression patterns in different clinicopathologic characteristics showed elevation in pathologic T stage, Histologic grade, Vascular invasion, AFP, Pathologic stage, and OS events subgroups (**Figures 6E–I**). However, no statistically significant differences were observed among HCC patients of different age, genders, BMI, and races (**Figures 6A–D**).

### 3.6 Prognostic value of SLC16A3 expression in HCC

**Figure 7:**
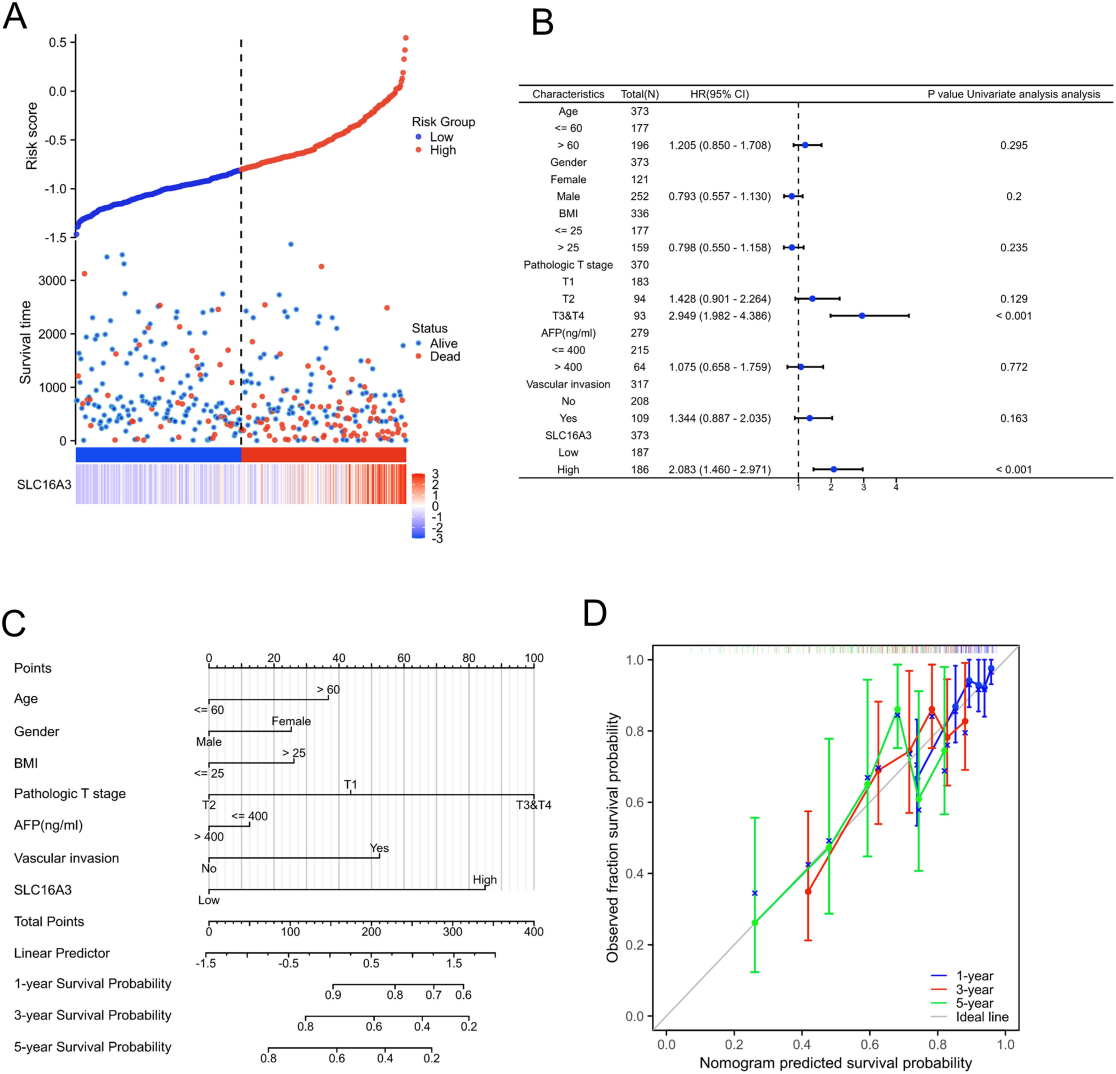
Prognostic value of SLC16A3 expression level in HCC. **(A)** SLC16A3 expression distribution and survival status. **(B)** Forest plot of OS by multivariate Cox regression analysis in HCC from TCGA database. **(C)** The nomogram for predicting 1-, 3-, or 5-year OS rates in patients with HCC. **(D)** The calibration curves for the nomogram.

We evaluated the prognostic significance of SLC16A3 expression in HCC patients within the TCGA database. First, an analysis of SLC16A3 expression levels, survival status, and risk scores demonstrated that elevated SLC16A3 expression in the high-risk group was associated with a higher mortality rate compared to the low-risk group (**Figure 7A**). Univariate Cox regression analysis revealed that increased SLC16A3 expression was linked to poorer OS (HR, 2.083; 95% CI, 1.460–2.971; *P* < 0.001). After adjusting for other clinicopathologic variables, multivariate Cox regression analysis confirmed SLC16A3 as an independent prognostic factor for HCC (HR, 1.946; 95% CI, 1.358–2.788; *P* < 0.001). Additional independent prognostic factors included age, gender, BMI, AFP levels, vascular invasion, and pathological T stage (**Figure 7B**). A nomogram model, integrating SLC16A3 expression and other independent prognostic variables identified through Cox regression analysis, was developed to predict OS at 1, 3, and 5 years (**Figure 7C**). Calibration curves were used to evaluate the nomogram’s predictive accuracy (**Figure 7D**).

### 3.7 Functional enrichment analysis of DEGs between SLC16A3 high and low expression groups in HCC

**Figure 8:**
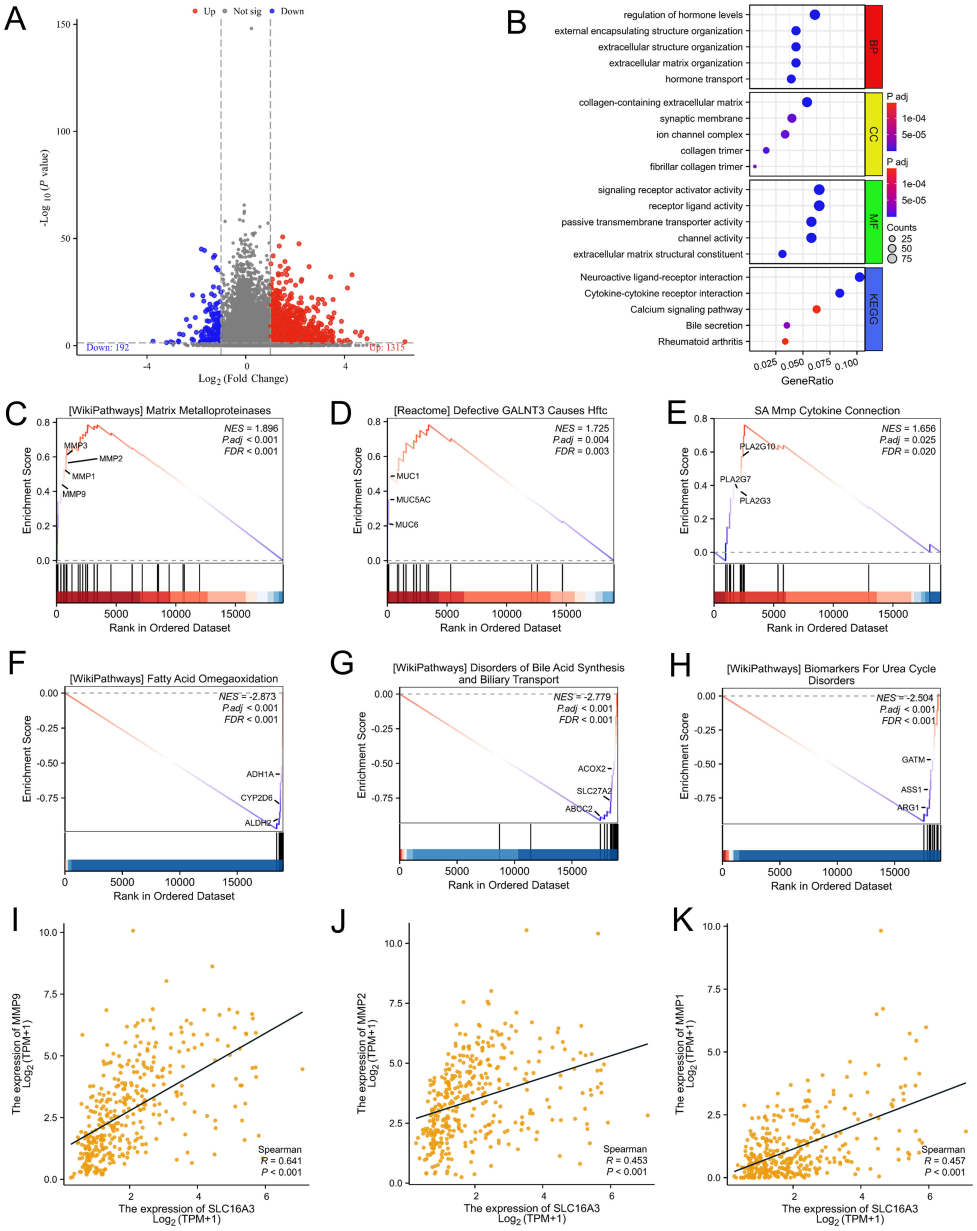
Functional enrichment analysis of DEGs between SLC16A3 high and low expression groups. **(A)** The volcano plot of DEGs. Red represents upregulated, blue represents downregulated genes. **(B)** GO and KEGG pathway enrichment analysis of DEGs. **(C–H)** GSEA functional enrichment analysis. **(I-K)** Scatterplots demonstrating the positive correlation of SLC16A3 expression with MMP9, MMP2, and MMP1. For correlation analyses, statistical significance was determined using Spearman’s correlation test. For enrichment analyses, *P* < 0.05 was considered statistically significant.

We analyzed DEGs between SLC16A3 high and low expression groups based on the median SLC16A3 expression level. A total of 1507 DEGs were identified, comprising 1315 upregulated and 192 downregulated genes (**Figure 8A**). In GO enrichment analysis, the biological process (BP) mainly contained regulation of hormone levels, extracellular matrix organization, and extracellular structure organization. The cellular component (CC) was enriched in collagen-containing extracellular matrix, collagen trimer, synaptic membrane, and fibrillar collagen trimer. The molecular function (MF) was mainly involved in receptor ligand activity, signaling receptor activator activity, and extracellular matrix structural constituent. KEGG pathway enrichment analysis indicated that SLC16A3 potential participation in the regulation of Neuroactive ligand-receptor interaction, and Calcium signaling pathway (**Figure 8B**). GSEA was applied to further investigate the biological functions of SLC16A3. Upregulation of SLC16A3 expression correlated with Matrix Metalloproteinases, including core enrichment genes: MMP1, MMP2, MMP9 (**Figure 8C**). Defective GALNT3 Causes Hftc, with core enrichment genes such as MUC1, MUC5AC, MUC6 were enriched in the SLC16A3 high-expression phenotype (**Figure 8D**). SLC16A3 also impacted pathways related to SA Mmp Cytokine Connection, including core genes PLA2G10, PLA2G7, PLA2G3 (**Figure 8E**). Additionally, pathways related to Fatty Acid Omegaoxidation, Disorders of Bile Acid Synthesis and Biliary Transport, and Biomarkers For Urea Cycle Disorders, were enriched. These pathways were negatively correlated with high SLC16A3 expression (**Figures 8F–H**). These findings suggested that SLC16A3 may play a role in HCC invasion and migration, making it a promising target for HCC treatment. Subsequently, we further studied the MMP family of important proteins associated with cancer invasion and metastasis, and found that MMP1, MMP2, and MMP9 were significantly positively correlated with SLC16A3 (**Figures 8I–K**), which further verified that SLC16A3 is an important part of invasion and metastasis function in HCC.

### 3.8 Effect of MCT4 on the MMP family-associated migration and invasion in HCC cells

**Figure 9:**
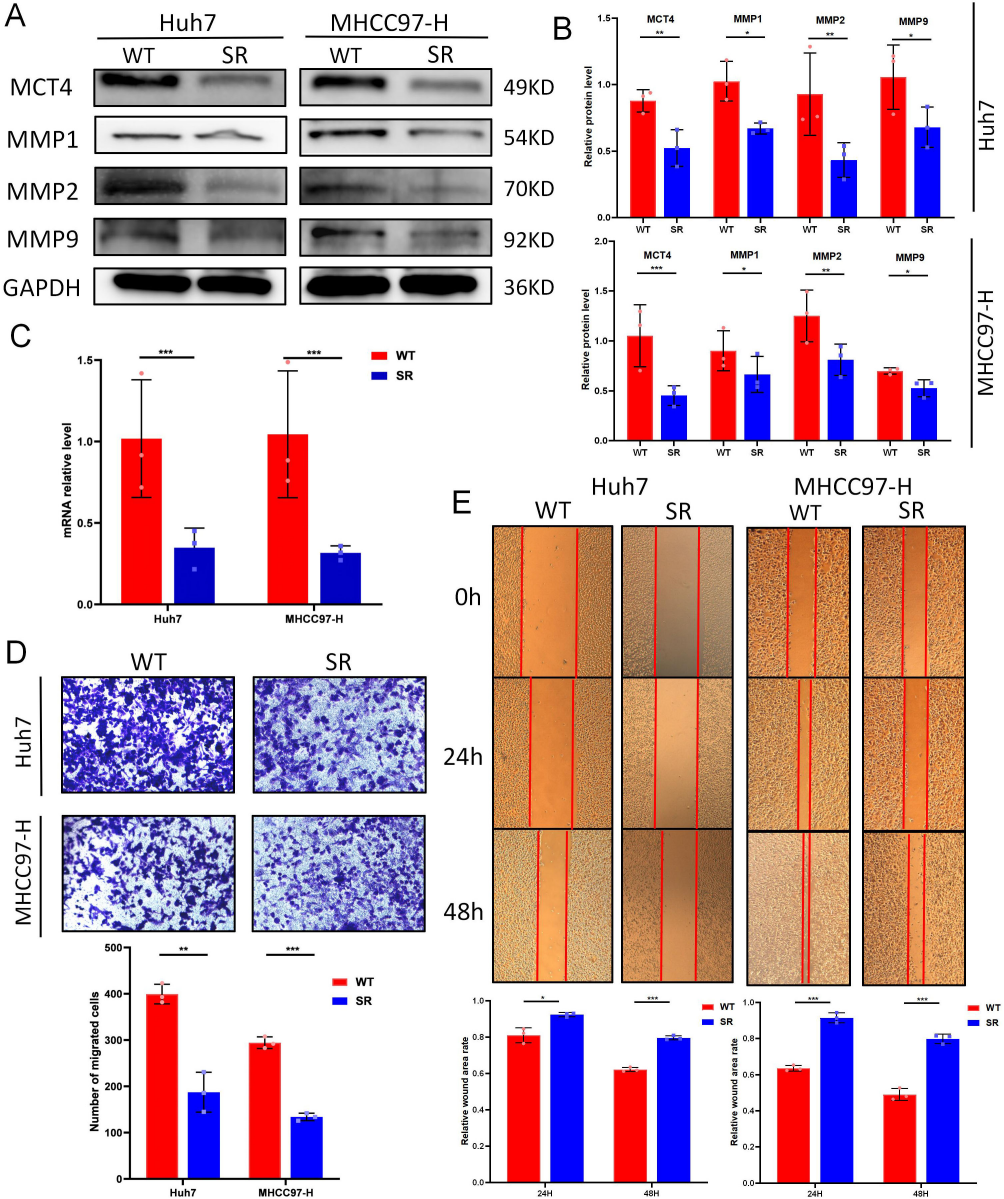
MCT4 knockdown suppresses MMP expression and inhibits HCC cell migration and invasion. **(A)** WB analysis of MCT4, MMP1, MMP2, and MMP9 protein expression in Huh7 and MHCC97-H cells. **(B)** Quantification of protein expression shown in panel **(A)**. **(C)** qPCR analysis confirming MCT4 knockdown efficiency at the mRNA level. **(D)** Transwell invasion assays and quantification. **(E)** Wound healing assays and quantification. Data are presented as mean ± SD from three independent experiments. Statistical significance was analyzed using an unpaired two-tailed Student’s t-test. ^*^P < 0.05, ^**^P < 0.01, ^***^P < 0.001. WT, wild-type/control group; SR, MCT4-knockdown group.

To investigate the role of MCT4 in HCC cells, we knocked down MCT4 in Huh7 and MHCC97-H cells. Western blot analysis showed that MCT4 protein expression was markedly reduced in the SR group compared with the WT group, and this was accompanied by decreased protein levels of MMP1, MMP2, and MMP9 in both cell lines (**Figures 9A, B**). RT-qPCR analysis further confirmed a significant reduction in MCT4 mRNA expression after knockdown (**Figure 9C**). Functionally, Transwell assays demonstrated that MCT4 knockdown significantly suppressed the invasive ability of Huh7 and MHCC97-H cells (**Figure 9D**). Consistently, wound healing assays showed that the migratory capacity of both cell lines was also markedly impaired after MCT4 knockdown, as reflected by a higher relative wound area at 24 h and 48 h in the SR group than in the WT group (**Figure 9E**). These findings indicate that MCT4 knockdown is associated with reduced MMP expression and decreased migratory and invasive capacities in HCC cells.

### 3.9 SLC16A3 (encoding MCT4) is associated with an immunosuppressive microenvironment in HCC

**Figure 10:**
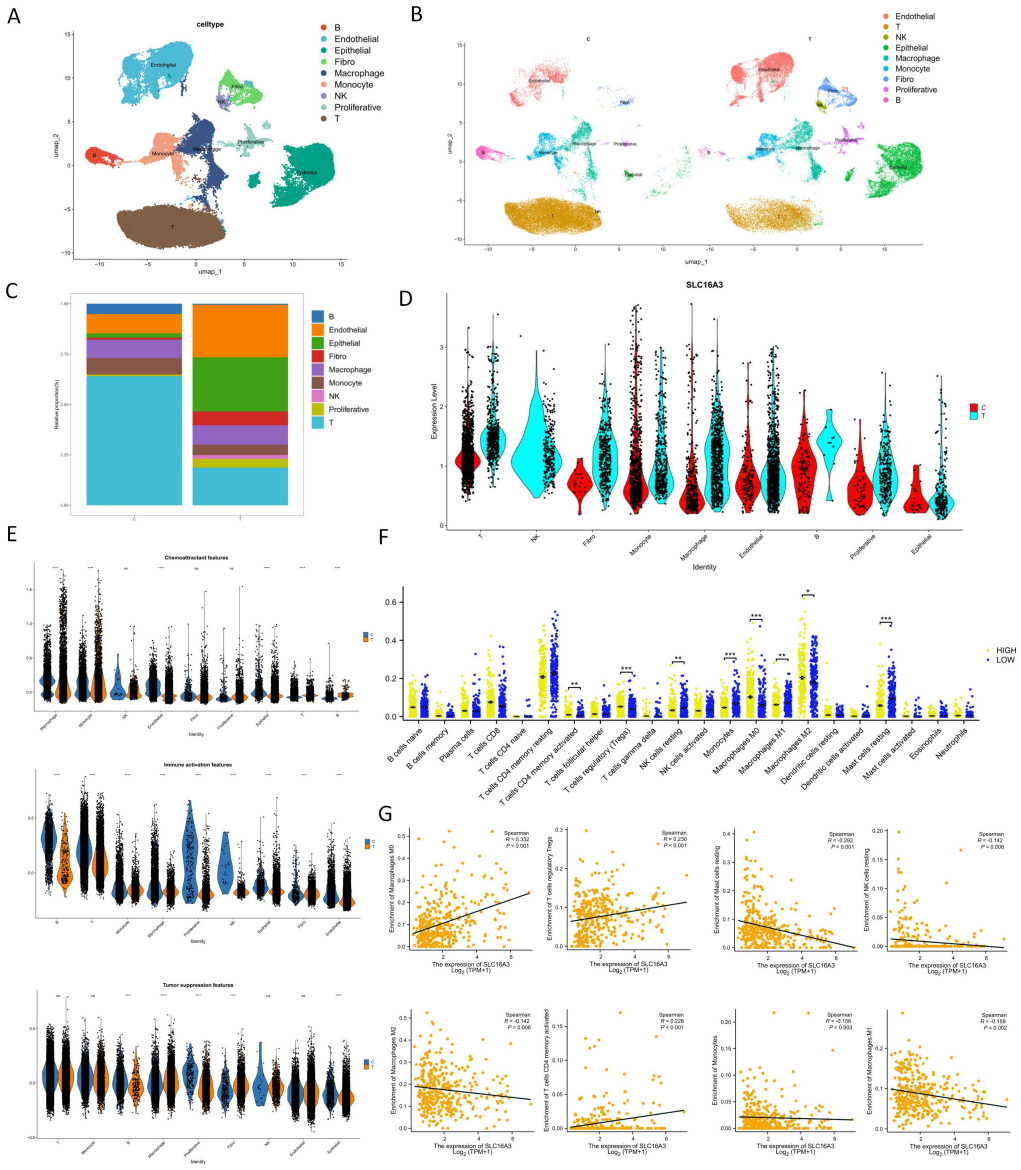
Single-cell RNA-seq analysis reveals the cellular distribution of SLC16A3 and its association with the immune microenvironment in HCC. **(A)** UMAP plot showing major cell populations identified in the single-cell dataset. **(B)** UMAP plots comparing cell distribution between adjacent non-tumor tissue (C) and tumor tissue (T). **(C)** Relative proportions of different cell populations in C and T groups. **(D)** Violin plots showing SLC16A3 expression across major cell types in C and T groups. **(E)** Violin plots showing differences in chemotactic, immune activation, and tumor suppression features among cell subsets between C and T groups. **(F)** Comparison of immune cell infiltration between SLC16A3 high and SLC16A3 low expression groups. **(G)** Correlation analysis between SLC16A3 expression and the infiltration levels of different immune cell types. For panels comparing two groups, statistical significance was analyzed using the Wilcoxon rank-sum test. Correlation analysis was performed using Spearman’s correlation test. ^*^P < 0.05, ^**^P < 0.01, ^***^P < 0.001, ^****^P < 0.0001, ns, not significant (*P* ≥ 0.05).

Based on single-cell RNA sequencing analysis of the GSE282701 dataset, clear differences in cellular composition were observed between HCC tissues and adjacent non-tumor tissues. UMAP clustering identified multiple major cell populations, including T cells, NK cells, macrophages, monocytes, endothelial cells, epithelial cells, fibroblasts, proliferative cells, and B cells (**Figure 10A**). Comparison between adjacent tissues and tumor tissues further demonstrated distinct distribution patterns of these cell populations (**Figure 10B**), and compositional analysis revealed marked differences in the proportions of immune and stromal cell subsets between the two groups (**Figure 10C**). Analysis of SLC16A3 expression at single-cell resolution showed that its expression varied across different cell types and differed between adjacent and tumor tissues, with relatively enriched expression detected in several immune and stromal populations, including macrophages (**Figure 10D**). Functional feature analysis further showed significant differences in chemotactic, immune activation, and tumor suppression-related signatures among cell subsets between adjacent and tumor tissues (**Figure 10E**). In addition, CIBERSORT analysis of bulk transcriptomic data indicated that the high-SLC16A3 expression group exhibited significantly altered immune infiltration patterns compared with the low-expression group, particularly in macrophage-related populations (**Figure 10F**). Correlation analysis further demonstrated that SLC16A3 expression was significantly associated with the infiltration of multiple immune cell subsets, especially macrophages, including M0 and M2 macrophages (**Figure 10G**). Together, these findings suggest that SLC16A3 is closely associated with remodeling of the immune microenvironment in HCC and may contribute to the formation of an immunosuppressive tumor niche.

### 3.10 MCT4 knockdown suppresses tumor growth and modulates macrophage polarization in a murine HCC model

**Figure 11:**
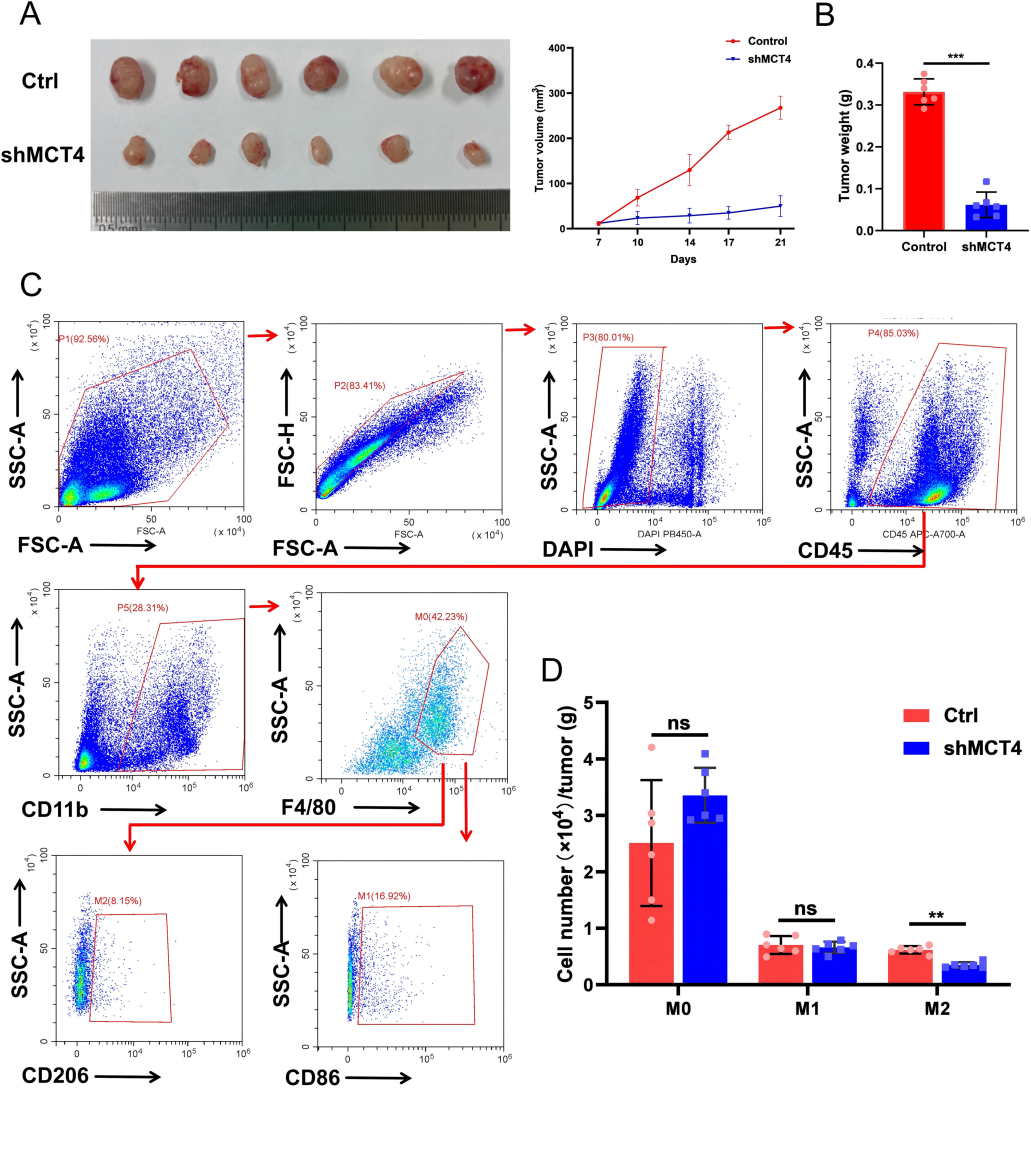
*In vivo* validation of the effects of MCT4 knockdown on tumor growth and macrophage polarization in HCC. **(A)** Representative images of subcutaneous tumors excised from the Ctrl and shMCT4 groups, and the corresponding tumor growth curves measured at the indicated time points. **(B)** Final tumor weights in the two groups. **(C)** Representative flow cytometry gating strategy for tumor-infiltrating macrophages. Nucleated cells were first gated based on FSC-A and SSC-A; single cells were selected using FSC-A and FSC-H to exclude doublets; live cells were identified as DAPI-negative cells; leukocytes were defined as CD45^+^ cells; myeloid cells were defined as CD11b^+^ cells; macrophages were identified as F4/80^+^ cells; M1 macrophages were defined as CD86^+^ cells and M2 macrophages as CD206^+^ cells. **(D)** Quantitative analysis of M0, M1, and M2 macrophage subsets in tumors from the two groups. Data are presented as mean ± SD. Statistical significance was analyzed using an unpaired two-tailed Student’s t-test. **P < 0.01, ***P< 0.001, ns, not significant; Ctrl, control group; shMCT4, MCT4-knockdown group.

To investigate the immunomodulatory role of MCT4 in HCC *in vivo*, we established a subcutaneous tumor model in mice using MCT4-knockdown Hepa1–6 cells. The results demonstrated that mice injected with MCT4-knockdown cells exhibited a significantly lower tumor growth rate compared to the control group (**Figure 11A**). Consistent with this, the weight of harvested subcutaneous tumors was significantly reduced in the shMCT4 group (**Figure 11B**). Flow cytometric analysis of tumor-infiltrating immune cells revealed no significant differences in M0 or M1 macrophage populations between the two groups. However, a marked reduction in CD206^+^ M2 macrophages was observed in the shMCT4 group (**Figures 11C, D**). Given the established role of M2 macrophages in restricting immune responses, promoting tumor angiogenesis, and facilitating tissue repair in HCC, these findings suggest that MCT4 inhibition attenuates CD206^+^ M2 macrophage polarization, thereby fostering a favorable immune microenvironment and impeding HCC progression.

## 4 Discussion

Studies have established that metabolic reprogramming and the TIME are two major factors contributing to therapeutic resistance in HCC (13, 14). This highlights the need for a deeper understanding of the intratumoral immune landscape in HCC to support more precise therapeutic strategies. Both metabolic reprogramming and the TIME are closely associated with the function of MCT4, which is primarily recognized as a lactate exporter. However, MCT4 has received relatively limited attention. With the emergence of the “lactate shuttle” concept (15, 16) and the discovery of lactylation (17–19), the critical role of lactate in various physiological and pathological processes has gradually become clearer (20), thereby renewing interest in MCT4. In the present study, we identified four lactate metabolism-related prognostic genes in HCC and performed a comprehensive analysis of the associations between these key genes and multiple immune cell types. We found that MCT4 was closely associated with the MMP family, which plays an important role in HCC invasion and metastasis, and that MCT4 significantly affected MMP expression as well as the migratory and invasive capacities of tumor cells. We also observed a strong association between MCT4 and tumor immunity. Integrated analysis of single-cell and bulk transcriptomic data suggested that MCT4 expression was highly correlated with macrophages and was associated with increased M2 macrophage polarization. Further investigation showed that MCT4 knockdown significantly suppressed HCC proliferation while concurrently reducing M2 macrophage polarization. Collectively, these findings suggest that MCT4 may serve as a potential therapeutic target in HCC.

Matrix metalloproteinases comprise a diverse family of enzymes with multiple physiological functions and are also involved in pathological processes such as cancer metastasis and inflammation (21). Specific MMPs, including MMP1 and MMP9, contribute to cancer progression by degrading extracellular matrix (ECM) components, thereby facilitating tumor invasion and metastasis (22). During tumor progression, MMP2 and MMP9 can promote tumor cell invasion through the basement membrane by degrading type IV collagen, thereby contributing to metastasis and dissemination (23). In addition, they may promote tumor growth by disrupting stromal barriers and enhancing angiogenesis (24). In the present study, GSEA revealed significant enrichment of MMP-related pathways in HCC patients with high MCT4 expression, suggesting that MCT4 may promote tumor progression and metastasis through the regulation of MMPs. We further validated this finding *in vitro* and showed that MCT4 knockdown in HCC cells significantly reduced the expression of MMP1, MMP2, and MMP9, accompanied by impaired migratory and invasive capacities. To our knowledge, this study provides the first experimental evidence supporting an association between the MCT4-MMP axis and HCC invasion and metastasis.

Previous studies have linked the lactate transporter MCT4 to the tumor immune microenvironment, particularly in breast cancer (25). Earlier studies generally evaluated MCT4 expression in bulk tumor tissues and examined its role in the broader tumor context. In breast cancer, MCT4 overexpression has been reported to promote tumor progression (26), and lactate has been shown to impair M1 macrophage polarization (27). Macrophages, as key components of the innate immune system, are the most abundant immune cell population in the tumor microenvironment (TME) of many solid tumors, including breast, colorectal, gastric, and lung cancers (28). Tumor-associated macrophages (TAMs) in highly aggressive solid tumors exhibit upregulated WNT signaling (29), which is involved in regulating the Warburg effect and glucose metabolism during tumor development (30). Notably, the TME can reprogram TAM metabolism through multiple mechanisms, including direct metabolite exchange, cytokine stimulation, and other signaling mediators (31). Certain TAM subsets actively promote tumorigenesis, angiogenesis, invasion, and therapeutic resistance, thereby contributing to poor clinical outcomes (32, 33). In our study, CIBERSORT analysis showed that MCT4 expression was associated with multiple immune cell populations in HCC, particularly M0, M1, and M2 macrophages. However, the scRNA-seq analysis did not show a significant difference in the overall proportion of macrophages between adjacent non-tumor tissue and tumor tissue, whereas epithelial cells and T cells displayed more prominent compositional differences. This apparent discrepancy may be attributable to the distinct analytical resolutions of the two approaches. Single-cell RNA-seq primarily reflects changes in the abundance of major cell populations, whereas CIBERSORT is more sensitive to transcriptional signatures associated with immune cell states and subtype transitions in bulk tissues. Therefore, the absence of a marked difference in total macrophage proportion does not exclude biologically meaningful changes in macrophage polarization. In particular, shifts between M1- and M2-like macrophage states may occur without a substantial change in total macrophage abundance. Moreover, the single-cell analysis remains informative because it confirms the presence of significant immune heterogeneity in HCC and demonstrates that MCT4 expression varies across different cell populations, including immune cells. Subsequent *in vivo* experiments further demonstrated that MCT4 knockdown reduced tumor volume and significantly decreased the proportion of M2 macrophages, suggesting that MCT4 may influence HCC progression through modulation of the macrophage-related immune microenvironment. Together, these findings support a close association between MCT4 and the immunosuppressive tumor microenvironment in HCC.

## 5 Conclusion

In summary, our study suggests that MCT4 is closely associated with HCC progression through two major aspects: it is linked to enhanced tumor invasion and metastasis in association with increased MMP expression, and it is associated with an immunosuppressive tumor microenvironment characterized by increased M2 macrophage abundance. These findings deepen our understanding of the potential role of lactate metabolism in HCC progression and highlight MCT4 as a potential therapeutic target. Future studies investigating MCT4 inhibitors, either alone or in combination with immunotherapy, may provide new strategies for improving HCC treatment outcomes.

## Data Availability

The data presented in this study were obtained from publicly available databases: GEO database, accession number GSE46408, GSE36411, GSE282701; TCGA-LIHC.
