# Supplementary material for: MCT4 drives HCC progression by activating MMPs and polarizing M2 macrophages
Source: Front Immunol. 2026 Jul 1;17:1777015. doi: 10.3389/fimmu.2026.1777015 (PMC13368753; doi:10.3389/fimmu.2026.1777015)
Supplement: Supplementary file 1 [file Table1.docx]

**Supplementary materials**

**Supplementary tables**

**Table S1. The sequences of siRNA used in this study**

| **shRNA** | **Sequence (5'-3')** |
| --- | --- |
| si-negative control | TTCTCCGAACGTGTCACGT |
| si-*MCT4-human* | TCATGCTTGTGGGGGGTCTCT |
| si-*MCT4-mouse* | CTGGGCTTCATCGACATCTTT |

**Table S2. Antibodies used in this study**

| **Antibody** | **Source** | **Identifier** | **Dilution (Application)** |
| --- | --- | --- | --- |
| MCT4/SLC16A3 (F2K6A) Rabbit mAb | CST | #81569 | 1:1000 (WB) |
| MMP-1 Rabbit Polyclonal Antibody | HUABIO | # ER31211 | 1:1000 (WB) |
| MMP-2 Recombinant Rabbit Monoclonal Antibody | HUABIO | # ET1606-4 | 1:1000 (WB) |
| MMP-9 Recombinant Rabbit Monoclonal Antibody | HUABIO | # ET1704-69 | 1:2000 (WB) |
| HRP-conjugated GAPDH Rabbit mAb | ABclonal | AC054 | 1:50000 (WB) |
| DAPI Solution | BD Pharmingen | 564907 | 1:1000 (FC) |
| Alexa Fluor® 700 Rat Anti-Mouse CD45 | BD Pharmingen | 560510 | 1:250 (FC) |
| FITC Mouse Anti-Rat CD11b | BD Pharmingen | 554982 | 1:100 (FC) |
| PE Rat Anti-Mouse F4/80 | BD Pharmingen | 565410 | 1:200 (FC) |
| PE-Cy™7 Rat Anti-Mouse CD86 | BD Pharmingen | 560582 | 1:200 (FC) |
| Alexa Fluor™ 647 Rat Anti-Mouse CD206 | BD Pharmingen | 568809 | 1:200 (FC) |
| HRP-conjugated Goat anti-Rabbit IgG (H+L) | ABclonal | Cat#AS014 | 1:10000 (WB) |
|  |  |  |  |

mAb, monoclonal antibody; pAb, polyclonal antibody; WB, western blot; FC, flow cytometer

**Table S3. Primers for RT-qPCR used in this study**

| **Gene Symbol** | **Gene Name** | **Primer Sequences（5'-3'）** |
| --- | --- | --- |
| *MCT4* | Monocarboxylate transporter 4 | F: ACTCCGTCTACCTCTTCAGC  R: ATGGCACTGGAGAACTTGTG |
| *ACTB* | actin beta | F: CATGTACGTTGCTATCCAGGC  R: CTCCTTAATGTCACGCACGAT |
